# Health seeking behaviour among Lebanese population: A highlight on seeking care from pharmacists

**DOI:** 10.1080/13814788.2021.1917541

**Published:** 2021-05-04

**Authors:** Rabih Soubra, Sani Hlais, Nadine Houmani, Lina Ghandour, Reda El Haj Hassan, Mohammed Joujou, Issam Shaarani

**Affiliations:** a Faculty of Medicine, Beirut Arab University, Beirut, Lebanon; b Faculty of Medicine, Saint Joseph University, Beirut, Lebanon

**Keywords:** Health-seeking behaviour, primary care, healthcare system, pharmacist, general practitioner

## Abstract

**Background:**

Understanding health-seeking behaviour could significantly reduce the impact of illness on patients’ lives. Fragmentation of the Lebanese healthcare system and presence of variability in socio-economic factors have affected some aspects of the Lebanese population’s overall health seeking behaviour. One of these aspects is seeking diagnosis from pharmacists, which is prohibited by the Lebanese law but reinforced by the absence of supervision of concerned authorities.

**Objectives:**

This study aimed to assess the Lebanese population’s knowledge, attitude, and practice towards seeking health care from pharmacists, exploring particularly the practice of seeking diagnosis from pharmacists.

**Methods:**

A cross-sectional study was conducted by surveying a convenient sample of 493 participants across the eight governorates of Lebanon between July and October 2016. A self-administered questionnaire was used. Questions assessed the health care seeking behaviour of the participants.

**Results:**

Two-thirds of the study participants (63.9%) did not have a general practitioner whom they visit regularly. Nearly half of the participants (48.9%) reported seeking diagnosis from pharmacists. Noteworthy, seeking diagnosis from pharmacists’ behaviour declined significantly with having a general practitioner visited regularly. More than half of participants (59.5%) believed that dealing with emergencies is among the pharmacists’ duties. In addition, 62.8% perceived that pharmacists are ‘often/always’ capable of managing common complaints.

**Conclusion:**

Our study showed that a significant proportion of the Lebanese population seek a diagnosis from pharmacists and a significant proportion of them have a misconception about the role of pharmacists in the Lebanese healthcare system.

KEY MESSAGES

- A significant proportion of the Lebanese population seek diagnosis from pharmacists
- Seeking diagnosis from pharmacists declines when having a primary care physician
- Raising awareness about proper roles in healthcare and providing accessible primary care are needed

## Introduction

Historically, Lebanon has suffered from a fragmented healthcare system because of long years of war and limited capability to recover afterwards [1]. Healthcare systems depend on the interaction between providers, recipients, and economic insurers to deliver the best healthcare services package [2]. In Lebanon, the private sector became a more significant provider of health care services given the poorly managed public sector. Around 42% of the Lebanese population do not have health coverage; hence, patients mainly pay the health services bills [1,3]. Besides, Lebanon lacks an official referral system with effective public primary healthcare services leading to an unorganised specialist-outpatient care directed by the patient’s health-seeking behaviour [4].

Health-seeking behaviour (HSB) refers to ‘any action or inaction undertaken by individuals who perceive themselves to have a health problem or to be ill for the purpose of finding an appropriate remedy’ [5]. HSB plays a pivotal role in establishing patients’ care plan and prognosis [6]. Appropriate HSB is a key to improve health outcomes and diminish morbidity and mortality rates.

In Lebanon, HSB can be influenced by many factors including social status, cost of services, gender issues, physical and psychological matters [7,8]. It’s believed that lacking health coverage affects the Lebanese population HSB [1]. For instance, Cheaito et al. [9] found that the main contributing factors behind self-medication in Beirut are lack of time and escape from paying medical doctor’s fees. In a study by Farah et al. [10], pharmacists provided over the counter antibiotics as per demand of self-medicating patients as they find a medical doctor’s visit expensive regardless of their socio-economic status.

The healthcare system pillars, including medical doctors, pharmacists, nurses and other health professionals, are responsible for delivering needed health care services with accountability. Pharmacists’ job has progressed far from manufacturing and dispensing prescribed medications [11]. Multifaceted obstacles confront community pharmacists in the Arab World and prevent them from playing their role in the community [12]. Nowadays, pharmacists are actively participating in the management plan and therapy adjustment. Interventions include patient health education and counselling, as well as medication and patient supervision. Thus, their role is complementary to the medical doctors’ role that facilitates proper collaborative care and guarantees better patients outcomes [11,13].

According to regulations promulgated by the Order of Pharmacists in Lebanon (OPL), pharmacists are not allowed to dispense any medication without a medical doctor prescription [14]. However, oversupply of pharmacists in Lebanon might be affecting their adherence to ethical and professional obligations and impair the quality of health service provided to the population [15]. For instance, a study in Beirut area showed that 32% of the antibiotics provided by pharmacists lacked a medical prescription in both high and low socio-economic areas leading to unnecessary antibiotics dispensation and poor health outcomes [10]. This emphasises the benefit of raising public awareness concerning the roles of each healthcare professional to achieve a successful and beneficial HSB with favourable outcomes on patients.

In Lebanon, no studies have yet tackled the extent to which Lebanese population seek healthcare from pharmacists as first-line healthcare providers and the factors that contribute to accepting this care. This study aims to assess the knowledge, attitude, and practice of the Lebanese population regarding healthcare seeking from pharmacists and seek diagnosis from pharmacists.

## Methods

### Study design and setting

This cross-sectional knowledge, attitude, and practice (KAP) study was conducted in the eight governorates of Lebanon: Beirut, Mount Lebanon, North, Akkar, Beqaa, Baalbek-Hermel, South, and Nabatiyeh (Supplementary Figure). Ethical approval was obtained from the Institutional Review Board of Beirut Arab University before conducting the study. Members of the research team, who are medical students at Beirut Arab University Faculty of Medicine, collected the data in COOP Association of Consumption and Production supermarket stores. COOP is a Lebanese supermarket franchise with stores distributed in the eight Lebanese governorates. People of different socio-economic status access COOP supermarket stores. Data collection lasted for four months, from July until October 2016.

### Recruitment and study participants

Data was collected during the opening hours of COOP, between 8:00 am and 10:00 pm, throughout the seven weekdays on settled fixed stations. People entering the supermarket or passing in front of adjacent streets’ gates were invited to participate in the study. Lebanese citizens who are at least 21 years old were recruited. Each participant signed an informed consent after a brief introduction about the study. Participants who accepted to participate filled a self-administered questionnaire.

### Questionnaire

An English version of the questionnaire was developed based on similar previous studies [16,17] and modified to fit the Lebanese situation. The Arabic version of the questionnaire was piloted on 50 people to collect feedback, check for misinterpretation of addressed points, and optimise its readability and validity. The questionnaire consisted of 30 multiple choice and Yes-No questions covering the sociodemographic background of participants and their HSB including health care seeking behaviour from pharmacists (Supplementary Material 1). The average time to fill the questionnaire was estimated to be 10 min.

### Sample size

The sample size was calculated using Creative Research Systems calculator version 2012 [18]. For a power of 80%, a margin of error of 5%, a confidence interval of 95%, and an estimated proportion of 50% to seek diagnosis from pharmacists, the calculated sample size was 384 participants as a minimum number.

### Statistical analyses

Data was entered and analysed using Statistical Package for the Social Sciences (SPSS, Chicago, IL) version 21.0. Descriptive statistics were inferred using simple cross tabulation, whereas the associations between dependent and independent variables were tested by chi-square. Logistic regression models were tested to study the effect of some variables on seeking diagnosis from pharmacists, with the latter serving as our dependent variable. Variables that were associated with seeking diagnosis from pharmacists in the bivariate analysis with a *p*-value of 0.2 or less were included in the model. Adjusted odds ratios with 95% confidence intervals were used for interpretation and reporting of results.

## Results

### Participants’ sociodemographic characteristics

Out of 710 individuals approached, 493 accepted to participate in the study, making the response rate 69.4%. The distribution of study participants with their sociodemographic factors is presented in Table 1. The study results revealed that more than 70% of participants were less than 50 years old. The Lebanese Force and Household Living Conditions Survey (LFHLCS) 2018–2019 [19] revealed similar percentages reinforcing that our sample was representative of the Lebanese population.

**Table 1.** Demographic information of the study participants and association with seeking diagnosis from pharmacists.

Demographic factors*	Seeking diagnosis from pharmacists			
	Total *N* = 493	Often/Always	No/Rarely	
	*n* (%)	*n* (%)	*n* (%)	*p*-Value
Age groups (years)				**0.021**
21–29	182 (37.1%)	94 (51.9%)	87 (48.1%)	
30–49	181 (36.9%)	98 (54.1%)	83 (45.9%)	
50–64	93 (18.9%)	37 (40.2%)	55 (59.8%)	
>65	35 (7.1%)	11 (31.4%)	24 (68.6%)	
Gender				0.147
Male	269 (54.6%)	123 (45.9%)	145 (54.1)	
Female	224 (45.4%)	117 (52.5%)	106 (47.5)	
Level of education				**0.017**
School education or less	207 (42.1%)	114 (55.1%)	93 (44.9%)	
College education	285 (57.9%)	125 (44.2%)	158 (55.8)	
Medical professionals				**0.003**
Health professionals	16 (4.5%)	2 (12.5%)	14 (87.5%)	
Non-health professionals	338 (95.5%)	172 (51%)	165 (49%)	
Average family income per month^a^				**0.001**
<500 USD	84 (17.1%)	48 (57.1%)	36 (42.9%)	
500–1000 USD	181 (36.9%)	95 (52.5%)	86 (47.5%)	
1000–2000 USD	156 (31.8%)	78 (50%)	78 (50%)	
>2000 USD	69 (14.1%)	18 (26.1%)	51(73.9%)	
Health insurance status				**0.002**
Insured	338 (68.8%)	149 (44.1%)	189 (55.9%)	
Not insured	153 (31.2%)	91 (59.5%)	62 (40.5%)	
Availability of a general practitioner visited regularly				**0.006**
Yes	177 (36.1%)	72 (40.7%)	105 (59.3%)	
No	313 (63.9%)	168 (53.7%)	145 (46.3%)	

*Total does not count always to 493 due to missing data; Rate of 1USD equals 1507.5LBP at the time of data collection; Marital status, governorate of residence, number of children in household, employment status, type of health coverage, coverage of prescribed drugs, and coverage of visit to doctor not represented as they were not significantly associated with the outcome variable

Bold values are significant *p*-values.

Among our study participants, 241 (48.9%) ‘often/always’ seek a diagnosis from pharmacists. A notable difference exists among the age groups as our results revealed that adult participants are more likely to seek diagnosis from pharmacists (around 50%) compared to elderly participants (31.4%) (*p* = 0.021). Being a healthcare professional was associated with significantly lower probability of seeking diagnosis from pharmacists with a *p*-value of 0.003 in the bivariate analysis, yet the association was not significant in the multivariate analysis. Notably, the three income groups of 2000 USD or less (1 USD equals 1507.5 LBP), ‘often/always’ seek diagnosis from pharmacists at similar percentages of around 50%; however, this percentage drops to 26% among the group whose participants’ average income is more than $2000 (*p* = 0.001). None of the demographic characteristics was a significant predictor of seeking diagnosis from pharmacists in the multivariate analysis.

### Participants’ knowledge outcome

Knowledge of participants regarding the laws set by OPL in Lebanon emphasising pharmacists’ duties is described in Table 2. Although around half of the study participants (45.1%) believe correctly that the OPL laws do not allow pharmacists to prescribe drugs, only 17.9% know that there is a law forbidding pharmacists to diagnose. Our results also showed that 90.4% know that diagnosing chronic diseases and prescribing a treatment regimen is not among the pharmacists’ roles.

**Table 2.** Knowledge of participants regarding the Order of Pharmacists in Lebanon (OPL) laws and the role of the pharmacists.

Assessment of knowledge	Total answered *n*	Answered correctly *n* (%)
OPL laws do not allow pharmacists to prescribe	492	222 (45.1%)
There is a law forbidding the Lebanese pharmacist to diagnose	492	88 (17.9 %)
Pharmacist should provide health care education and advise safe use of medications	491	348 (70.9%)
Pharmacist should formulate and distribute drugs	491	218 (44.4%)
Pharmacist should dispense drugs based on a medical doctor’s prescription	491	438 (89.2%)
Pharmacist should not diagnose chronic diseases and prescribe a treatment regimen	491	444 (90.4%)
Pharmacist should not dispense drugs without prescription	491	437 (89.0%)
Pharmacist should not suture lacerations	491	422 (85.9%)
Pharmacist should not deal with health emergencies (such as injuries, burns…etc.)	491	292 (59.5%)

**Table 3.** Unadjusted and adjusted Odds Ratios of seeking diagnosis from pharmacists by some demographic and healthcare coverage variables.

Variables	Unadjusted OR	95% CI for unadjusted OR	*p*-Value*	Adjusted OR	95% CI for adjusted OR	*p*-Value**
Age						
21–29	1	–	0.021	1	–	0.08
30–49	1.09	(0.72, 1.65)		1.01	(0.57, 1.77)	
50–64	0.62	(0.37, 1.03)		0.56	(0.29, 1.07)	
>65	0.42	(0.19, 0.91)		0.32	(0.08, 1.20)	
Gender						
Male	1	–	0.14	1	–	0.51
Female	1.30	(0.91, 1.85)		1.17	(0.71, 1.92)	
Education						
School education or less	1	–	0.017	1	–	0.51
College education	0.64	(0.45, 0.92)		0.84	(0.52, 1.41)	
Governorate						
Beirut	1	–	0.125	1	–	0.31
North	1.18	(0.66, 2.08)		1.41	(0.68, 2.92)	
South	1.29	(0.73, 2.27)		1.68	(0.80, 3.52)	
Beqaa	0.90	(0.50, 1.63)		0.78	(0.36, 1.69)	
Mount Lebanon	0.60	(0.32, 1.12)		0.74	(0.34, 1.62)	
Nabatieh	0.65	(0.35, 1.22)		1.04	(0.46, 2.35)	
Job health professionals						
Health professionals	1	–	0.003	1	–	0.06
Non-health Professionals	7.29	(1.63, 32.6)		4.45	(0.92, 21.50)	
Income						
<500 USD	1	–	0.001	1	–	0.10
500–1000 USD	0.82	(0.49, 1.39)		0.77	(0.37, 1.59)	
1000–2000 USD	0.75	(0.43, 1.27)		0.96	(0.44, 2.10)	
>2000 USD	0.26	(0.13, 0.52)		0.36	(0.13, 0.97)	
Medical insurance						
Insured	1	–	0.002	1	–	0.08
Not insured	0.53	(0.36, 0.79)		0.63	(0.37, 1.07)	
Availability of a general practitioner visited regularly						
No	1	–	0.006	1	–	0.02
Yes	0.59	(0.40, 0.85)		0.57	(0.35, 0.93)	

*p-Value for bivariate analysis; **p-value for multivariate analysis.

### Participants’ attitude outcome

Although around half of the study participants (49.2%) think that visiting the pharmacist before the physician might worsen their case, nearly two-thirds (62.8%) of subjects included in our study think that pharmacists are ‘often/always’ capable of managing common complaints and around half are satisfied with the pharmacists’ diagnoses alone (56.3%). Around half of the population (46.2%) prefer consulting pharmacists rather than physicians to spare paying the consultation fees. Almost 46% consider that pharmacists know more about drugs’ side effects than physicians do. Accordingly, around one third (33.8%) support legalising drug prescription by pharmacists.

**Figure 1.**
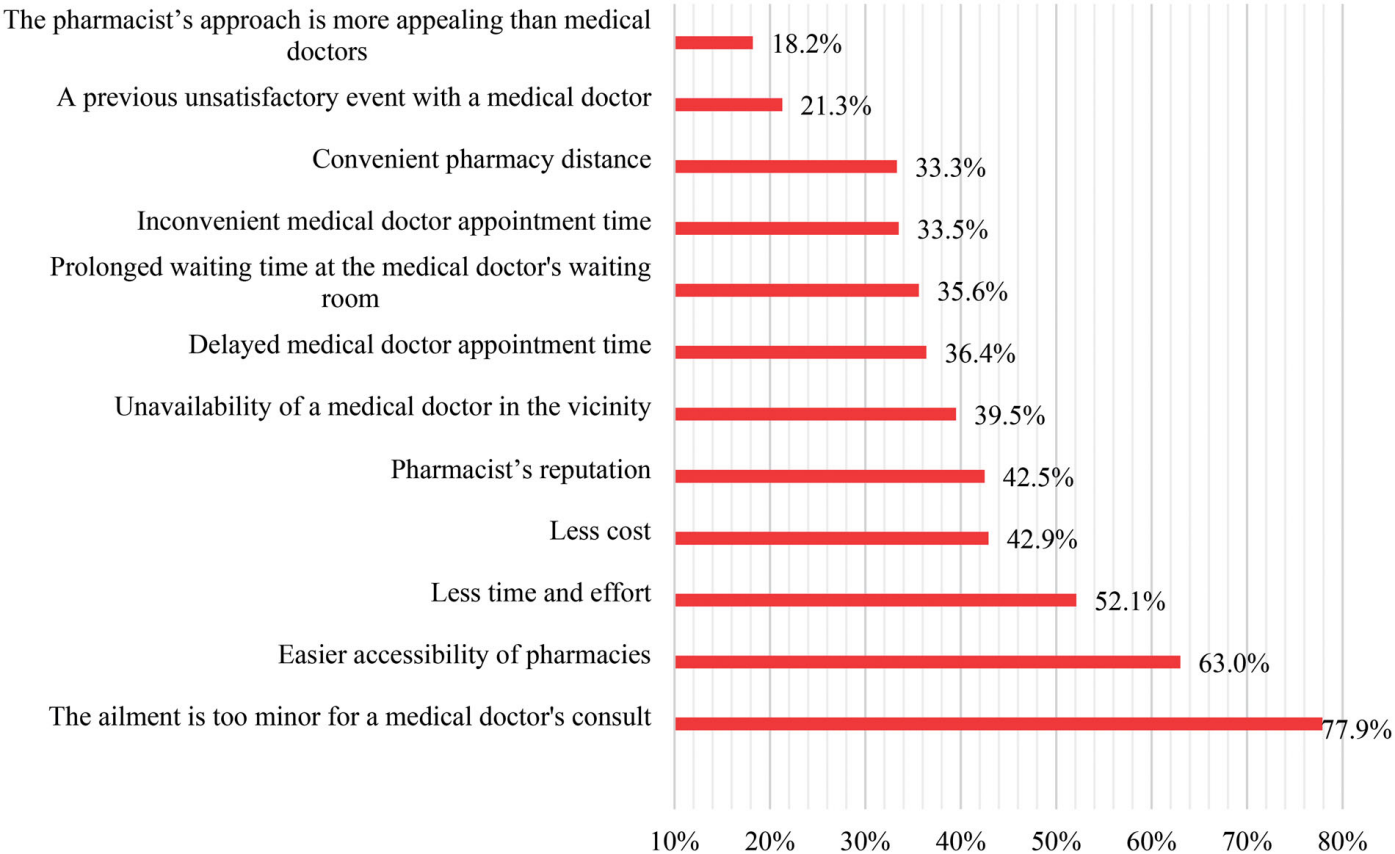
Factors influencing decision to seek medical care from pharmacist (*N* = 493**). *Total does not count always to 493 due to missing data. **Participants were able to select all that apply.

The perceived little seriousness of a complaint/sickness for a physician’s consult is the most important factor that encourages the population to seek pharmacists for a diagnosis (77.9%). Around two-thirds (63.0%) of the Lebanese population are influenced by easier accessibility of pharmacies and nearly half of them (52.1%) are affected by the time and effort saved by seeking pharmacists rather than a physician for health care (Figure 1).

### Participants’ practice outcome

Health insurance status significantly affected participants’ behaviour in seeking diagnosis from pharmacists. Around 44% of the study participants who have insurance seek diagnosis from pharmacists (149 of 338), whereas among the uninsured participants this number increased to around 60% (91 of 153) (*p* = 0.002).

Those who have a general practitioner visited regularly, seek diagnosis from pharmacists less frequently than those who do not have one (*p* = 0.006). The adjusted OR of seeking diagnosis from pharmacists among participants who reported having a general practitioner regularly visited was 0.57, 95% CI (0.35, 0.93) (Table 3).

**Figure 2.**
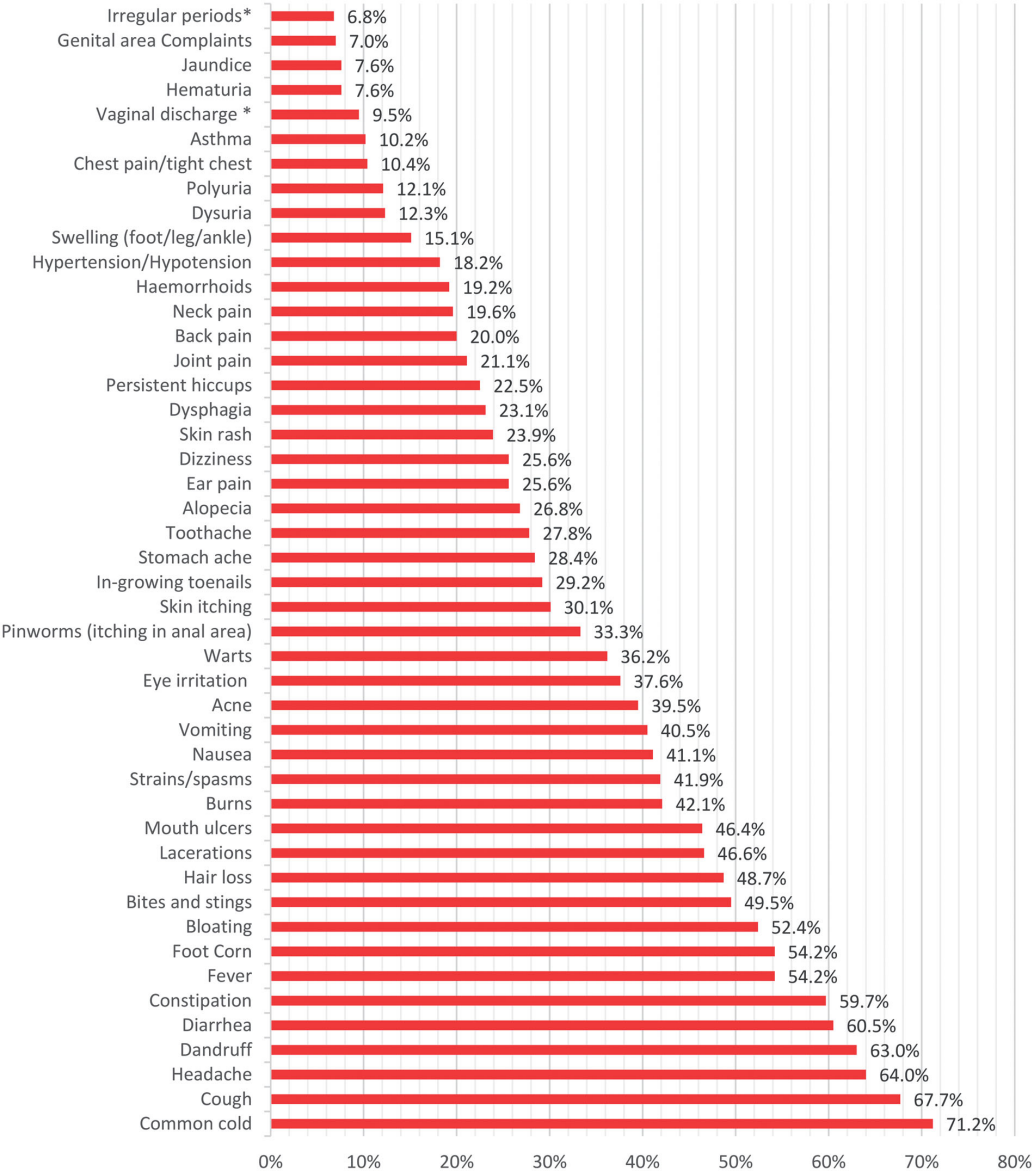
Conditions for which pharmacists were visited to get health care (*N* = 493**). *Conditions apply to females only. **Total does not count always to 493 due to missing data

The most common conditions for which participants consult pharmacists included common cold, cough, headache, diarrhoea while participants were less likely to consult pharmacists for urogenital complaints, jaundice, asthma, chest pain; as showed in Figure 2.

## Discussion

### Main findings

In exploring the various factors affecting the Lebanese population behaviour in seeking diagnosis from pharmacists rather than a medical doctor, we found that those who visit a general practitioner regularly, those who are between 21 and 49 years old, those who have health insurance, those who are health professionals, and those with an average monthly family income higher than $2000 had a significantly lower odds of seeking pharmacists for health care, particularly in obtaining a diagnosis.

### Interpretation of the study – The results in relation to existing literature

Our study showed that seeking diagnosis from pharmacists was less among participants who have a general practitioner regularly visited than those who do not have. This highlights the importance of having access to a general practitioner. Similarly, a study held in 31 European countries showed that availability of a general practitioner and sustained communication between the practitioner and patients significantly increases their propensity to seek his medical care for major and minor complaints [20].

On the other hand, it was noted that delayed medical doctor’s appointment and prolonged waiting time spent at medical doctor’s office affected 36.4% and 35.6% of the participants correspondingly to seek health care from pharmacists instead of medical doctors. Similarly, in a study held in Qatar, necessity of a medical doctor’s appointment and long waiting time in clinic pushed around 76% and 78% of the participants respectively to seek pharmacists rather than medical doctor for diagnosis and treatment [21].

Other factors influencing the decision to seek health care from pharmacists as confirmed by our participants were easier accessibility of pharmacies (63%) and less cost (42.9%). Correspondingly, easier accessibility (86%) and lower fees (46%), were major factors for health care seeking behaviour from pharmacists in a study conducted in Bangladesh highlighting the management of respiratory illnesses by pharmacists [22].

Added to that, approximately two-thirds of the participants preferred visiting pharmacists for minor complaints such as common cold, cough, and headache. Similarly, two-thirds of participants in a study in Poland would seek pharmacists’ advice for flu and/or flu-like symptoms. On the contrary, in United Kingdom (UK), most participants confirmed that they wouldn’t visit a pharmacist if they had a minor complaint [23].

By law and according to the Lebanese Ministry of Public Health, pharmacists’ duties include manufacturing medicines as well as dispensing prescribed and over the counter (OTC) medications [14]. Only 17.9% of the participants in our study knew that there is a Lebanese law forbidding pharmacists from giving diagnosis to patients. This vague perception of the Lebanese law could be explained by the incomplete understanding of pharmacists’ duties by people. Another two major causes of this misconception could be the educational status and medical knowledge among the participants. Forty-two percent (42%) of our participants have at best-reached school education level and 95.5% are non-health professionals. A study conducted in Pakistan supports our assumption where participants were confused about the role of pharmacists and what they are authorised to do; most participants (60%) had high-school degree only [24].

In addition, around one-third of the participants in our study think that dealing with emergencies is among the pharmacists’ duties and responsibilities. This raises concerns regarding this practice and the possible poor management that pharmacists might provide in such emergencies. Affirmatively, a study in Australia showed that pharmacists lacked the skills of epinephrine injection procedures in managing anaphylaxis emergencies, although they were aware about the symptoms of anaphylaxis and the urgent need of ambulatory care [25].

In a nutshell, an integrated healthcare system highlighting the role of primary healthcare centres with appropriate referral system would enhance the national healthcare management [20]. Cooperation between healthcare providers ensures proper medical care in the presence of adequate resources, appropriate planning, and patient’s assistance [26]. This sets the seal on the importance of the complementary inter-professional collaboration between pharmacists and medical doctors to fulfil the best patient care [27].

### Strengths and limitations

This study is one of the few studies that addressed healthcare seeking behaviour of the Lebanese population, highlighting seeking diagnosis from pharmacists in a comprehensive way. Our study started back in 2016 and it explores the aspects of seeking health care from pharmacists and reveals many misconceptions and improper practices regarding HSB. The COVID-19 pandemic imposed safety regulations to stop viral transmission. The latter has contributed to a considerable proportion of patients visiting pharmacies instead of physicians, especially for minor illnesses. This is thought to happen mainly due to easier accessibility and availability of local pharmacies during lockdown [28]. Our study findings can help address these practices while developing policies and procedures for the current situation. It is an eye-opener and a trigger for further research about this topic.

Among the limitations encountered during this research was that the concept of ‘seeking medical care’ in the questionnaire might have been perceived in different ways by participants. In addition, our sampling was suboptimal as we did not have sufficient resources to conduct household sampling. Some questions might have been prone to recall bias, as questions were general in nature and not related to a recent encounter with pharmacists. Also noteworthy is that due to the numerous questions included in our questionnaire some participants omitted to answer certain questions, subsequently reflected by lack of total response in certain variables.

### Implications for research and practice

A significant proportion of the Lebanese population would seek diagnosis from pharmacists with most of them lacking a defined primary care physician, highlights the rising need for providing accessible and affordable primary care services for the entire population. Furthermore, the results demonstrate a requisite to raise awareness about the different roles designated for pharmacists and medical doctors in the healthcare system. Also, further training for community pharmacists on managing common and minor complaints is required to optimise therapy.

## Conclusion

Our study results illustrated that many Lebanese citizens would seek diagnosis from pharmacists, and a significant proportion of them have a misconception about the role of pharmacists in the Lebanese healthcare system. Patients who have a general practitioner, whom they visit regularly, are less likely to seek diagnosis from pharmacist.

## Supplementary Material

Final Questionaire 6 (arabic version) (v.6.0)

Final Questionaire 6 (english version) (v.6.0)

Supplementary Figure
