# Supplementary material for: Health seeking behaviour among Lebanese population: A highlight on seeking care from pharmacists
Source: Eur J Gen Pract. 2021 May 4;27(1):51–9. doi: 10.1080/13814788.2021.1917541 (PMC8816400; doi:10.1080/13814788.2021.1917541)
Supplement: Final Questionaire 6 (arabic version) (v.6.0) [file IGEN_A_1917541_SM6230.pdf]

مجلس أخلاقيات البحث العلمي

استمارة الموافقة المستنيرة

العنوان: طلب العناية الطبية: المعرفة والموقف والممارسة بين السكان اللبنانيين .

الباحث الرئيسي: د.عصام الشعراني والسيد ربيع سوبرة.

التاريخ: ٢٠١٦/٠٦/٢٧

هدف الدراسة البحثية:
إن هذا البحث يهدف إلى التحقق من طريقة طلب العناية الطبية عند المواطن اللبناني وتقييم ومعرفة أسباب توجهه إلى الصيدلي كمصدر أوّلي عند الحاجة للعناية الطبية.
نتوقع مشاركة ٤٨٠ لبنانياً في هذه الدراسة.

الاجراءات:
يُطلب من المشارك تعبئة استمارة استطلاع علما بأن ذلك لن يأخذ من وقته أكثر من عشرة دقائق.

المخاطر/العوائق:
إن المخاطر المقترنة بالمشاركة في هذه الدراسة لا تتعدى تلك التي نواجهها في الحياة اليومية أو خلال الاستفسار الروتيني عن تاريخك الصحي.

الفوائد:
إن المشاركة في هذه الدراسة لا تعود عليكم بأي فوائد مباشرة. من الممكن أن تُفيد هذه الدراسة المجتمع اذا ما أدت نتائجها الى تقييم وكشف العوامل التي تؤثر على أفراد المجتمع اللبناني في اختيار الصيدلي كمصدر أوّلي للعناية الطبية.

المشاركة الطوعية و حق الإنسحاب:
إن مشاركتكم في هذه الدراسة هي مشاركة طوعية بالكامل حيث أن قرار المشاركة يعود لكم وهو بمحض إرادتكم. في حال قررتم عدم المشاركة فلن يترتب عليكم أية عقوبات ولن تخسروا أي من الإمتيازات التي تحق لكم.
وفي حال إختيار المشاركة في الدراسة، يمكن لكم التوقف عن المشاركة في أي وقت دون أي عقوبات أو فقدان أي من الإمتيازات المستحقة. عند اختيار التوقف عن المشاركة في الاستبيان، يرجى طلب ذلك من الشخص الذي يملأ الإستبيان.
إن قرار الانسحاب من الدراسة لا يرتب أية عقوبات تذكرعلى المشارك.

الظروف التي قد تؤدي الى إنهاء مشاركتكم
في ظل ظروف معينة، قد نقرر انهاء مشاركتك في الدراسة قبل استكمال تعبئة استمارة الاستبيان. وعلى وجه التحديد، فإننا قد نضع حداً لمشاركتك اذا كنت لا تتمتع بالشروط والمواصفات المطلوبة.

السرية

سيتم الحفاظ على سرية سجلات الدراسة الّتي تُعرَف عنكم. تجدر الإشارة الى أنه قد يقوم أشخاص مسؤولون عن جودة البحث العلمي بمراجعة سجلات مشاركاتكم في البحث لضمانة سلامة الاجراءات البحثية المتبعة، بما في ذلك أعضاء مجلس أخلاقيات البحث العلمي في جامعة بيروت العربية. وفيما عدا ذلك، فإن الاطلاع على السجلات الخاصة بكم لن يكون متاحا سوى للأشخاص القائمين على الدراسة، ما لم تعطوا إذنا لغيرهم للإطلاع على سجلات الدراسة.

من أجل الحفاظ على سرية المعلومات، سوف يتم استعمال أرقام مرمزة بدلا من الاسماء الحقيقية وسوف يتم وضع السجلات في صندوق مقفل. لن يتم كشف أي معلومات لأي شخص كان أو لأي طرف كان ما عدا الأشخاص القائمين على الدراسة.

التعويض:

عند المشاركة في هذه الدراسة لن تتلقى أية مكافأة مالية أو أي تعويض آخر.

إذا كانت لديك أية أسئلة أو إستفسارات:

يمكنكم طرح الأسئلة حول هذه الدراسة الأن أو في أي وقت أثناء إجراء الدراسة، وذلك من خلال التحدث الى الباحث أو الباحثين الذين يعملون معكم أو من خلال الاتصال بالدكتور عصام الشعراني ٠٣\١٣٥١١٠ أو السيد ربيع سوبرة ٠٣\٨٣١٨٨٥.

إذا كانت لديك أية أسئلة بخصوص حقوقك كمشارك في البحث أو في حال تعرضك لأي معاملة غير منصفة الرجاء الإتصال بمجلس أخلاقيات البحث العلمي في جامعة بيروت العربية على الرقم ٠٠٩٦١١٣٠٠١١٠ مقسم ٢٧٤٣ .

ما يترتب عليه توقيعكم:

إن توقيعكم في أسفل الصفحة يعني أنكم قد فهمتم المعلومات الواردة في إستمارة الموافقة المستنيرة. كما يعني أيضا موافقتكم على المشاركة في الدراسة.

إن توقيعكم على إستمارة الموافقة لا يعد تنازلا عن أية حقوق قانونية قد تحصلون عليها من جراء مشاركتكم في هذه الدراسة البحثية.

توقيع المشارك:

التاريخ:

توقيع الشخص الحاصل على الموافقة:

التاريخ:

ID# ______

الجزء الأول: (معلومات عامة)

١) العمر : ______

٢) الجنس:

١- ذكر ٢- انثى

٣) السكن:

١- بيروت ٢- الشمال ٣- الجنوب

٤- البقاع ٥- جبل لبنان ٦- النبطية

٤) المستوى التعليمي:

١- غير متعلم ٢- ابتدائي ٣- متوسط

٤- ثانوي ٥- جامعي ٦- دراسات العليا

٥) الحالة الاجتماعية؟

١- أعزب ٢- متأهل ٣- مطلق ٤- ارمل

٦) هل لديك أولاد:

١- لا ٢- نعم (حدد العدد) _____

٧) أ- ما هي وظيفتك الحالية:

١- لا أعمل ٢- اعمل(حدد طبيعة العمل) ______________ (إنتقل إلى ٨)

ب- إذا كنت لا تعمل، أنت حالياً:

١- تلميذ (حدد الإختصاص) ______________ ٢- ربة منزل ٣- متقاعد

٤- عاطل عن العمل ٥- غير قادر على العمل (ذوي الحاجات الخاصة) ٦- لم يتم ذكره (حدد) ______________

٨) متوسط الدخل الشهري للأسرة:

١- أقل من ٧٥٠،٠٠٠ ل.ل
٢- بين ٧٥٠،٠٠٠ ل.ل و ١،٥٠٠،٠٠٠ ل.ل
٣- بين ١،٥٠٠٠،٠٠٠ ل.ل و ٣،٠٠٠،٠٠٠ ل.ل
٤- أكثر من ٣،٠٠٠،٠٠٠ ل.ل

٩) أ- هل لديك تغطية صحية/تأمين؟

١- لا (إنتقل إلى **١٠**)
٢- نعم

ب- إذا كانت الإجابة نعم، حدد نوع التغطية: (يمكنك إختيار أكثر من إجابة)

١- تعاونية موظفي الدولة
٢- الصندوق الوطني للضمان الاجتماعي
٣- تأمين الجيش
٤- تأمين الأمن الداخلي
٥- شركة تأمين خاصة
٦- أخرى (حدد) ___________

ج- ماذا يغطي التأمين الصحي الخاص بك (حدد نسبة التغطية):

(١) زيارة الطبيب ١- لا ٢- نعم _____ (%)

(٢) الأدوية الموصوفة ١- لا ٢- نعم _____ (%)

<u>الجزء الثاني:(المعرفة-المواقف-الممارسات)</u>

١٠) عندما تمرض ماذا تفعل عادةً؟

١- أستشير الطبيب
٢- أستشير الصيدلي
٣- أداوي نفسي
٤- شيء آخر (حدد) _____________

١١) هل لديك طبيب رعاية صحية تقوم بزيارته بإنتظام؟

١- لا
٢- نعم

١٢) هل تؤثر رسوم الإستشارة الطبية (الكشفية) على قرارك بزيارة الطبيب؟

١- أبداً ٢- نادراً ٣- أحياناً ٤- دائماً

١٣) هل تقوم بزيارة الصيدلي للحصول على تشخيص لحالتك المرضية؟

١- أبداً ٢- نادراً ٣- أحياناً ٤- دائماً

١٤) رأيك، هل الصيدلي قادر على معالجة الأمراض الشائعة؟

١- أبداً ٢- نادراً ٣- أحياناً ٤- دائماً

١٥) هل ينصحك الصيدلي باستشارة الطبيب في بعض الحالات؟

١- أبداً ٢- نادراً ٣- أحياناً ٤- دائماً

١٦) هل تكون راضياً بالتشخيص الذي يقدمه الصيدلي، وإن لم يتم تأكيده من قبل الطبيب؟

١- أبداً ٢- نادراً ٣- أحياناً ٤- دائماً

١٧) هل تعتقد أن زيارتك للصيدلي قبل الطبيب قد تساهم في تفاقم/تدهور حالتك الصحية؟

١- أبداً ٢- نادراً ٣- أحياناً ٤- دائماً

١٨) هل تقوم بالاتصال هاتفيا بالصيدلي للحصول على تشخيص؟

١- أبداً ٢- نادراً ٣- أحياناً ٤- دائماً

١٩) اذا نصحك الصيدلي بتناول دواء لم يصفه لك طبيبك، هل تقوم بتناوله؟

١- لا ٢- نعم

٢٠) هل يسمح القانون اللبناني للصيدلي بوصف الأدوية؟

١- لا ٢- نعم ٣- نعم، لكن في حالات معينة فقط

٢١) هل يقوم الصيدلي بمراجعة طبيبك عند تغييره للدواء الموصوف؟

١- أبداً ٢- نادراً ٣- أحياناً ٤- دائماً

٢٢) اذا قام الصيدلي بتغيير الدواء الذي وصفه الطبيب لك، هل تأخذه؟

١- لا ٢- نعم ٣- نعم، لكن بعد مراجعة الطبيب

٢٣) برأيك، هل يعرف الصيدلي الآثار الجانبية للأدوية أكثر من الطبيب؟

١- لا ٢- نعم

٢٤) هل توافق على جعل وصف الأدوية من قبل الصيدلي قانونياً؟

١- لا ٢- نعم

٢٥) ما موقفك من هذا التّصريح: " يبالغ الصيدلي احياناً بوصف الأدوية للمريض بغية بيع المزيد من الأدوية"

١- أوافق وبشدة ٢- أوافق ٣- لا أوافق ٤- لا أوافق وبشدة

٢٦) هل تظن أنّ هناك قانونا في لبنان يمنع الصيادلة اللّبنانيين من تشخيص حالات المرضى؟

١- لا ٢- نعم ٣- لا أعلم

٢٧) ماذا يفعل معظم الصيادلة الّذين تقوم بزيارتهم؟ (يمكنك إختيار أكثر من إجابة)

١- تشخيص الأمراض ووصف الأدوية وفقا لتشخيصه
٢- صرف الدواء تبعًا لوصفة الطّبيب فقط
٣- بيع الأدوية الّتي لا تحتاج لوصفة طبية من دون الاستفسار عن الحالة المرضية
٤- بيع الأدوية التي تحتاج لوصفة طبية دون سؤالك عن الوصفة

٢٨) بحسب معرفتك، ما هي وظيفة الصيدلي؟ (يمكنك إختيار أكثر من إجابة)

١- توفير التوعية الصحيّة وتقديم النصائح حول الإستخدام الآمن للأدوية
٢- تركيب الأدوية وصرفها
٣- تشخيص الأمراض المزمنة ووصف العلاج
٤- صرف الأدوية بناءَ على الوصفة الطّبيّة
٥- إعطاء الحقن بناءَ على الوصفة الطّبيّة
٦- إعطاء اللّقاحات بناءَ على الوصفة الطّبيّة
٧- صرف معظم الأدوية دون وصفة طبيّة
٨- إعطاء الحقن دون وصفة طبيّة
٩- إعطاء اللّقاحات دون الوصفة الطّبيّة
١٠- تقطيب الجروح
١١- التّعامل مع الحالات الطارئة (مثل الجروح والحروق... إلخ)

٢٩) هل تؤثر العوامل التالية على قرارك للحصول على العناية الطبية من الصيدلي؟ (ضع علامة ✓ في المكان المناسب)

العوامل	نعم	لا
١- كوْن إستشارة الصيدلي مجانية		
٢- توفر الإستشارة والدواء معاً		
٣- عدم توفر طبيب في محيط منطقتي		
٤- سهولة الوصول الى الصيدلية في أي وقت		
٥- أوقات مواعيد الطبيب لا تناسبني		
٦- موعد الطبيب بعيد زمنياً		
٧- فترة الانتظار عند الطبيب طويلة		
٨- طريقة الصيدلي في مقاربة حالتي المرضية أفضل من الأطباء		
٩- المسافة إلى الصيدلية أقرب من المسافة الى عيادة الطبيب		
١٠- شهرة الصيدلي المهنية		
١١- لحالة المرضية بسيطة لا تحتاج لإستشارة الطبيب		
١٢- تجربة سابقة غير مريحة مع الطبيب		

٣٠) في أي من الحالات التالية تزور الصيدلي؟ (ضع علامة ✓ في المكان المناسب)

الحالة		نعم	لا
١- الصداع			
٢- ارتفاع الحرارة			
٣- الرشح			
٤- السعال			
٥- إرتفاع أو إنخفاض ضغط الدم			
٦- ألم في الأذن			
٧- ألم في الاسنان			
٨- حساسية في العين (إحمرار العين)			
٩- حالات الربو			
١٠- قروح في الفم (حمو)			
١١- ألم في المعدة			
١٢- ألم في الصدر أو ضيق في النفس			
١٣- ألم في الظهر			
١٤- ألم في المفاصل			
١٥- ألم في الرقبة			
١٦- تشنج في العضلات			
١٧- كثرة التبول			
١٨- حرقة في البول			
١٩- دم في البول			
٢٠- إفرازات مهبلية غير إعتيادية	للنساء فقط		
٢١- دورة شهرية غير منتظمة			
٢٢- أمراض الأعضاء التناسلية			
٣٣- ديدان			

الحالة	نعم	لا
٢٤- قشرة في الرأس		
٢٥- تساقط الشعر		
٢٦- الثعلبة (شعر الرأس)		
٢٧- مسمار القدم		
٢٨- ظفر غارس في اللحم		
٢٩- حب الشباب		
٣٠- الطفح الجلدي		
٣١- حكاك في الجلد		
٣٢- الحروق		
٣٣- الجروح		
٣٤- الثآليل (تواليل)		
٣٥- لسعات ولدغات الحشرات		
٣٦- الحازوقة		
٣٧- الصعوبة في البلع		
٣٨- الغثيان (اللعيان)		
٣٩- نفخة في البطن (غازات)		
٤٠- التقيؤ (الاستفراغ)		
٤١- الإسهال		
٤٢- الإمساك (الإكتام)		
٤٣- الدوخة		
٤٤- اليرقان/الصفيرة (إصفرار العين)		
٤٥- البواسير		
٤٦- تورم الأرجل		
