# Supplementary material for: Health seeking behaviour among Lebanese population: A highlight on seeking care from pharmacists
Source: Eur J Gen Pract. 2021 May 4;27(1):51–9. doi: 10.1080/13814788.2021.1917541 (PMC8816400; doi:10.1080/13814788.2021.1917541)
Supplement: Final Questionaire 6 (english version) (v.6.0) [file IGEN_A_1917541_SM6229.pdf]

Informed Consent Form

Title: Seeking Medical Care: Knowledge, Attitude and Practice amongst the Lebanese Population.

PI: Dr. Issam Shaarani and Mr. Rabih Soubra

Date: 27/06/2016

PURPOSE OF RESEARCH STUDY:

The aim of this research is to assess the health-seeking behaviours of the Lebanese population and their tendencies to acquire medical care/diagnosis from pharmacists as the primary source.
We anticipate that approximately 480 Lebanese will participate in this study.

PROCEDURES:

The participant will be given a questionnaire to fulfil that take no longer than 10 minutes.

RISKS/DISCOMFORTS:

The risks associated with participating in this study do not exceed those that we face in daily life or during the performance of routine history taking.

BENEFITS:

There are no direct benefits coming back to you by participating in this study. This study can benefit the community if the results led to the acknowledgment of the factors that are affecting the Lebanese population's visit to the pharmacist as a primary health care source.

VOLUNTARY PARTICIPATION AND RIGHT TO WITHDRAW:

Your participation in this study is entirely voluntary: You choose whether to participate. If you decide not to participate, there are no penalties, and you will not lose any benefits to which you would otherwise be entitled.
If you choose to participate in the study, you can stop your participation at any time, without any penalty or loss of benefits. If you want to withdraw from the study, you have to state your wish for the person filling the questionnaire.
The decision to withdraw from the study will not have any significant consequences for the participant.

CIRCUMSTANCES THAT COULD LEAD US TO END YOUR PARTICIPATION:

Under certain circumstances we may decide to end your participation before you have completed the questionnaire. Specifically, we may stop your participation if we find out that you do not fit the criteria set for this study.

CONFIDENTIALITY:

Any study records that identify you will be kept confidential. The records from your participation may be reviewed by people responsible for making sure that the research is done properly, including members of the BAU Institutional Review Board. (All these people are required to keep your identity confidential). Otherwise, records that identify you will be available only to people working on the study, unless you give permission for other people to see the records.

The use of code numbers rather than participants' names on data sheets and keeping records in a locked file cabinet will be done to protect confidential information. Aside from the team members running the study, the information collected will not be disclosed to anyone else or other parties.

COMPENSATION:

You will not receive any payment or other compensation for participating in this study.

IF YOU HAVE QUESTIONS OR CONCERNS:

You can ask questions about this research study now or at any time during the study, by talking to the researcher(s) working with you or by calling Dr. Issam Shaarani on 03/135110 or Mr. Rabih Soubra on 03/831885.

If you have questions about your rights as a research participant or feel that you have not been treated fairly, please call the BAU Institutional Review Board at 00961 1 300110 ext. 2743.

WHAT YOUR SIGNATURE MEANS:

Your signature below means that you understand the information in this consent form. Your signature also means that you agree to participate in the study.

By signing this consent form, you have not waived any legal rights you otherwise would have as a participant in a research study.

Participant's Signature:

Date:

Signature of Person Obtaining Consent:

Date:

ID# _____

Part I: (General info)

1) Please specify your age: _____

2) State your gender:

   1- Male   2- Female

3) In which district of Lebanon do you live:

   1- Beirut   2- North   3- South
   4- Beqaa   5- Mount Lebanon   6- Nabatieh

4) What is the highest level of education that you have completed?

   1- No education   2- Elementary School   3- Middle School
   4- Secondary School   5- Undergraduate   6- Postgraduate

5) What's your marital status:

   1- Single   2- Married   3- Divorced   4- Widowed

6) Do you have any children?

   1- No   2- Yes (specify number) _____

7) A- Current employment status:

   1- Not working   2- Yes (specify job) __________ (skip **B**)

   B- If not working you are?

   1- a student (specify specialty) __________
   2- a housewife
   3- Retired
   4- Unemployed
   5- Unable to work (Disabled)
   6- Not mentioned (specify), __________

8) Average family's income per month:

   1- < 750.000 L.L   2- 750,000 L.L – 1,500,000 L.L
   3- 1,500,000 L.L - 3,000,000 L.L   4- > 3,000,000 L.L

9) A- Do you have health coverage/insurance?

1- No (skip **B** & **C**)   2- Yes

B- If yes, specify: (choose all that applies)

1- COOP   2- NSSF   3- Military Health insurance

4- Internal Security Forces   5- Private insurance   6- Others (specify) ___________

C- What does your medical insurance cover (mention the percentage covered):

(1) Physician visit   1- No   2- Yes _____ (%)

(2) Prescribed drugs   1- No   2- Yes _____ (%)

Part II: (Knowledge-Attitude-Practice)

10) What do you usually do when you get sick?

1- Consult a physician   2- Consult a pharmacist

3- Self-medicate   4- Others (Specify) ___________

11) Do you have a primary health care physician whom you visit regularly?

1- No   2- Yes

12) Does the consultation fee affect your decision to consult a physician?

1- Never   2- Rarely   3- Often   4- Always

13) Would you visit the pharmacist to get a diagnosis?

1- Never   2- Rarely   3- Often   4- Always

14) In your opinion, is the pharmacist able to manage common ailments?

1- Never   2- Rarely   3- Often   4- Always

15) Does your pharmacist advise you to consult a physician in certain cases?

1- Never   2- Rarely   3- Often   4- Always

16) Would you be satisfied with a pharmacist's diagnosis alone, without confirming it with a physician?

1- Never 2- Rarely 3- Often 4- Always

17) Do you think visiting a pharmacist before visiting a physician, might worsen your case?

1- Never 2- Rarely 3- Often 4- Always

18) Would you call a pharmacist for diagnosis?

1- Never 2- Rarely 3- Often 4- Always

19) If your pharmacist advised you to take a drug that your doctor didn't prescribe, would you take it?

1- No 2- Yes

20) Does the Lebanese law allow pharmacists to prescribe?

1- No 2- Yes 3- Yes, but only in certain conditions

21) Does your pharmacist review your physician upon changing a prescribed drug?

1- Never 2- Rarely 3- Often 4- Always

22) If your pharmacist changed the medication that your physician prescribed, will you take it?

1- No 2- Yes 3- Yes, but after checking with my physician

23) In your opinion does a pharmacist know more about drug's side effects than a physician does?

1- No 2- Yes

24) Do you agree with legalizing the prescription of drugs by the pharmacist?

1- No 2- Yes

25) What is your attitude towards this statement: "A pharmacist may sometimes overprescribe drugs as he/she has interest in selling more drugs" .

1- I strongly disagree 2- I disagree 3- I agree 4- I strongly agree

26) Do you think there is a law that forbids the Lebanese pharmacists to diagnose?

1- No
2- Yes
3- I don't know

27) What does most of the pharmacists you visit do: (You can pick more than one answer)

1- Diagnose and prescribe drugs based on their diagnosis
2- Dispense drugs based on physician's prescription only
3- Sells you the OTC drug you came for without questioning
4- Sells you prescription drug without asking about the physician's prescription

28) According to your knowledge, what is the role of the pharmacist: (You can pick more than one answer)

1- Provide health care education and advise safe use of medications
2- Formulate and distribute drugs
3- Diagnose chronic diseases and prescribe a treatment regimen
4- Dispense drugs based on a physician's prescription
5- Administer injections based on a physician's prescription
6- Administer vaccines based on physician's prescription
7- Dispense most drugs without prescription
8- Administer injections without physician's prescription
9- Administer vaccines without physician's prescription
10- Suture lacerations
11- Deal with emergencies (such as injuries, burns…etc)

Part III: (Factors - Medical conditions)

29) Do the following factors influence your decision to seek medical care from a pharmacist? (Place a ✓ in the corresponding box)

Factors	Yes	No
1- Less cost (free consultation)		
2- Less time and effort (simultaneous availability of consultation and medication)		
3- Unavailability of a physician in the vicinity of my region		
4- Easier accessibility of pharmacies (more convenient time,might stay for night, opens 24 hrs)		
5- Inconvenient physician appointment time		
6- Delayed physician appointment time		
7- Prolonged waiting time at the physician's waiting room		
8- The pharmacist's approach is more appealing than physicians		
9- Convenient pharmacy distance (pharmacy is closer to my house than the physician's clinic)		
10- Pharmacist's Reputation		
11- The ailment/sickness is too minor for a physician's consult		
12- A previous unsatisfactory event with a physician		

30) For which of the following conditions would you visit a pharmacist? (Place a ✓ in the corresponding box)

**Condition**		**Yes**	**No**
1- Headache			
2- Fever			
3- Common cold			
4- Cough			
5- Hypertension/Hypotension			
6- Ear pain			
7- Toothache			
8- Eye irritation (eye redness)			
9- Asthma			
10- Mouth ulcers			
11- Stomach ache			
12- Chest pain/tight chest			
13- Back pain			
14- Joint pain			
15- Neck pain			
16- Strains/spasms			
17- Excess urination			
18- Burning urine			
19- Blood in urine			
20- Vaginal discharge	Skip if male		
21- Irregular period			
22- Genital area diseases			
23- Worms (itching in anal area)			

**Condition**	**Yes**	**No**
24- Dandruff		
25- Hair loss		
26- Alopecia		
27- Corn		
28- In-growing toenails		
29- Acne		
30- Skin rash		
31- Itchy skin		
32- Burns		
33- lacerations		
34- Warts		
35- Bites and stings		
36- hiccups		
37- Difficulty in swallowing		
38- Nausea		
39- Bloating		
40- Vomiting		
41- Diarrhea		
42- Constipation		
43- Dizziness		
44- Jaundice (yellow eyes)		
45- Haemorrhoids		
46- Swelling (foot/leg/ankle)		
