# Supplementary material for: Health seeking behaviour among Lebanese population: A highlight on seeking care from pharmacists
Source: Eur J Gen Pract. 2021 May 4;27(1):51–9. doi: 10.1080/13814788.2021.1917541 (PMC8816400; doi:10.1080/13814788.2021.1917541)

**Supplementary Figure.** **Illustrated map demonstrating the eight governorates of Lebanon (Beirut, Mount Lebanon, North, Akkar, Beqaa and Baalbek-Hermel, South, and Nabatieh) and the recruitment distribution of corresponding areas.**


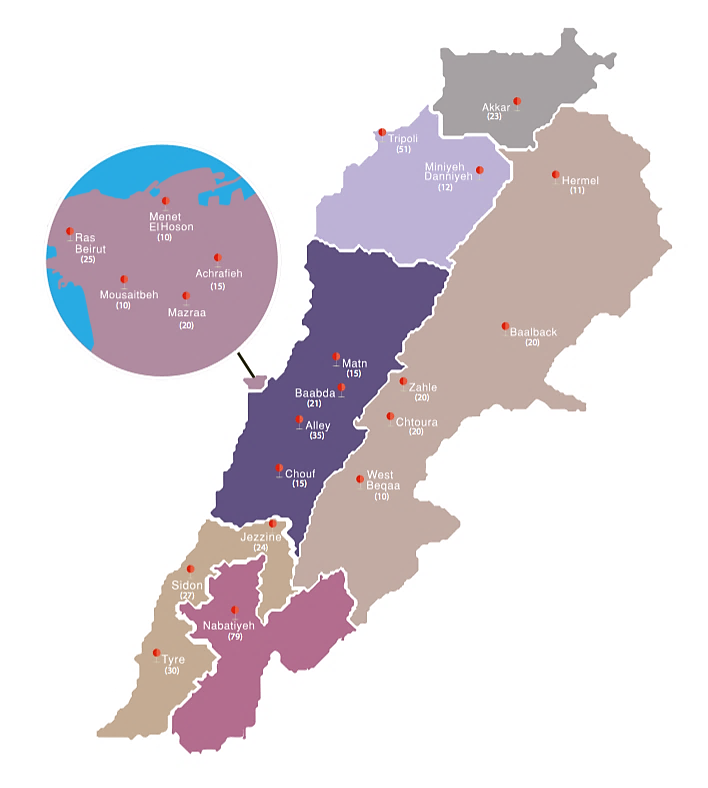

Supplement: Supplementary Figure [file IGEN_A_1917541_SM6228.docx]
